# Asking about self-harm during risk assessment in psychosocial assessments in the emergency department: questions that facilitate and deter disclosure of self-harm

**DOI:** 10.1192/bjo.2023.32

**Published:** 2023-05-25

**Authors:** Rose McCabe, Clara Bergen, Matthew Lomas, Mary Ryan, Rikke Albert

**Affiliations:** Division of Health Services Research and Management, City University of London School of Health Sciences, London, UK; Department of Biosciences, University of Exeter, Exeter, UK; Health Systems Innovation Lab, London South Bank University, London, UK; East London NHS Foundation Trust, London, UK

**Keywords:** Suicide, risk assessment, emergency department, communication, conversation analysis

## Abstract

**Background:**

Emergency departments are key settings for suicide prevention. Most people are deemed to be at no or low risk in final contacts before death.

**Aim:**

To micro-analyse how clinicians ask about suicidal ideation and/or self-harm in emergency department psychosocial assessments and how patients respond.

**Method:**

Forty-six psychosocial assessments between mental health clinicians and people with suicidal ideation and/or self-harm were video-recorded. Verbal and non-verbal features of 55 question–answer sequences about self-harm thoughts and/or actions were micro-analysed using conversation analysis. Fisher's exact test was used to test the hypothesis that question type was associated with patient disclosure.

**Results:**

(a) Eighty-four per cent of initial questions (*N* = 46/55) were *closed* yes/no questions about self-harm thoughts and/or feelings, plans to self-harm, potential for future self-harm, predicting risk of future self-harm and being okay or keeping safe. Patients disclosed minimal information in response to closed questions, whereas open questions elicited ambivalent and information rich responses. (b) All closed questions were *leading*, with 54% inviting no and 46% inviting yes. When patients were asked no-inviting questions, the disclosure rate was 8%, compared to 65% when asked yes-inviting questions (*P* < 0.05 Fisher's exact test). (c) Patients struggled to respond when asked to predict future self-harm or guarantee safety. (d) Half of closed questions had a narrow timeframe (e.g. at the moment, overnight) or were tied to possible discharge.

**Conclusion:**

Across assessments, there is a bias towards not uncovering thoughts and plans of self-harm through the cumulative effect of leading questions that invite a no response, their narrow timeframe and tying questions to possible discharge. Open questions, yes-inviting questions and asking how people feel about the future facilitate disclosure.

Suicide prevention is a global public health priority, with over 700 000 deaths by suicide annually.^1^ Communicating with people in distress about suicide is highly sensitive. Meta-analysis indicates that only 28.7% of people disclose suicidal intent to healthcare professionals before suicide.^2^ Many patients are reported to ‘deny suicidal ideation’ in the days or weeks before a suicide attempt.^3,4^ Importantly, UK professionals judge immediate risk of suicide to be low or not present in 85% of assessments in the final appointment before death.^5^ Self-harm, self-injury or self-poisoning irrespective of intent^6^ is the strongest risk factor for suicide.^7^ Most studies have focused on professional reports or patients’ medical records rather than direct research on communication about self-harm.

## Communicating about self-harm

Communicating about self-harm is challenging. Sociological studies have shown a strong preference for ‘saving face’ and not disclosing face-threatening and stigmatised information in social interactions.^8^ Self-harm remains highly stigmatised, and patients report shame, guilt, fear about loss of autonomy^9^ and onward consequences such as admission or removal of children from their care.^10^

Recent studies have highlighted that clinicians’ questions about self-harm can have a considerable impact on disclosure of thoughts and plans. Yes/no questions are prevalent in medical interaction. Although medical communication training encourages professionals to ask non-leading questions, there is no such thing as a non-leading yes/no question. Yes/no questions communicate an expectation in favour of either ‘yes’ or ‘no’ responses through wording and grammatical structure,^11^ e.g. ‘Are you feeling suicidal?’ is framed positively, inviting agreement to ‘feeling suicidal’.^12^ Conversely, ‘Not feeling suicidal?’ is negatively framed, inviting agreement to ‘not feeling suicidal’. Word choice further reinforces the direction of the question. Words such as ‘any’, ‘ever’, ‘at all’ are negative polarity items (e.g. ‘Any suicidal thoughts?’) that invite a ‘no self-harm’ response and affect patients’ likelihood of responding ‘no’.^13^

In primary care, most questions invite ‘no self-harm’ answers, with patients showing difficulty reporting self-harm thoughts and/or actions when clinicians ask these leading questions (e.g. ‘You're not feeling suicidal?’).^14^ In secondary out-patient mental healthcare, more than 75% of questions invited patients to confirm the absence of self-harm thoughts and/or actions.^15^ Patients were significantly more likely to say no in response to no-inviting questions (compared with yes-inviting questions), even though some disclosed suicidal thoughts in self-report measures.

## Psychosocial assessments in the emergency department

A key setting for suicide prevention is the emergency department:^6^ around 220 000 episodes of self-harm by 150 000 people are managed by emergency departments in England annually.^16^ Most emergency departments have a psychiatric liaison team staffed by specialist mental health clinicians. They conduct psychosocial assessments after self-harm and make onward referrals.^6^ Emergency departments are high-pressure environments with 4 h targets for patients to be seen, treated and admitted or discharged. Although the number of people seeking help for mental health problems has risen year on year, numbers of hospital beds have fallen,^17^ increasing the pressure to discharge patients. To date, no studies have observed psychosocial assessments for self-harm in emergency departments. This study addressed the following questions.

- How do clinicians ask patients about suicidal ideation and/or self-harm in emergency department psychosocial assessments?
- How do patients respond verbally and non-verbally?

## Method

### Study design

This was a cross-sectional non-participant observational study of clinical practice, involving video-recording psychosocial assessments between people presenting to the emergency department for suicidal ideation and/or self-harm and psychiatric liaison clinicians.

### Setting

The study took place in an emergency department in England. As per usual practice, people seeking care for suicidal ideation and/or self-harm met with emergency department triage staff to assess clinical urgency and undergo any physical interventions before referral to the emergency department liaison psychiatry service for a psychosocial assessment, including risk of harm to self^6^, providing the basis for a decision on whether to discharge and a management plan for further support.

### Ethics

All procedures contributing to this work comply with the ethical standards of the relevant national and institutional committees on human experimentation and with the Helsinki Declaration of 1975, as revised in 2008. All procedures were approved by London Central Research Ethics Committee (17/LO/1234). Study design, materials and recruitment processes were developed in collaboration with a lived experience group including one carer, one mental health nurse and six people who had presented to the emergency department for suicidal ideation and/or self-harm. Written informed consent was obtained from all participants.^18^

### Participants

All 43 psychiatric liaison clinicians in the psychiatric liaison team who conducted psychosocial assessments were invited to participate, and 33 consented (76.7%). Clinician participants comprised mental health nurses (*N* = 11), a student nurse (*N* = 1), doctors (*N* = 2), junior doctors (*N* = 7), social workers (*N* = 2), occupational therapists (*N* = 2), healthcare assistants (*N* = 2), consultant psychiatrists (*N* = 2), psychiatry trainees (*N* = 2) and unspecified (*N* = 2). Their mean age was 38.3 years (s.d. 11.6, range 23–62), and they were mostly female (*N* = 20/33) and White British (*N* = 28/33).

Patients referred for a psychosocial assessment for suicidal ideation and/or self-harm were approached by a clinician who assessed capacity to give informed consent. Exclusion criteria were: age under 16, cognitive difficulties, active psychosis, requiring an interpreter or subject to a restriction order.

Recruitment took place from September 2018 to April 2019. A total of 260 referrals were screened, 82 individuals were approached regarding participation and 48 consented. Three patients were excluded after consenting, as they did not present with suicidal ideation or self-harm. One patient re-presented, resulting in a total of 46 assessments. Referrals were for suicidal ideation (*N* = 20/46), self-harm by overdose (*N* = 23/46) or self-harm by ligature or attempted drowning (*N* = 3/46). Patient mean age was 35.5 years (s.d. 15, range 18–71). They were mostly female (*N* = 31/45) and White British (*N* = 43/45). Caregivers, typically parents, were involved in eight assessments.

### Video data

Forty-six psychosocial assessments with 45 patients and 23 clinicians were video-recorded.^18^ Two GoPro cameras were placed in the room with no researcher present.

### Data analysis

All recordings were reviewed to identify clinicians’ initial questions about:

- current thoughts and/or feelings about self-harm, e.g. ‘Are you having any thoughts that you'd be better off not here?’
- acting on thoughts or repeating self-harm, e.g. ‘If you went home now. Would you do anything to harm yourself?’
- plans to self-harm, e.g. ‘Do you have any plans to do anything to harm yourself?’

We focused on initial questions about thoughts, actions and plans of self-harm, typically asked in the middle of the assessment within a series of risk assessment questions. We did not examine follow-up questions (e.g. ‘And how long have you been feeling this way?’) as the focus was on initial disclosure of thoughts, actions or plans. Questions about acting on thoughts or repeating self-harm were typically asked at the end of the assessment, alongside questions about next steps and discharge.

We used a qualitative method, conversation analysis,^19^ to micro-analyse clinicians’ questions and patients’ responses. Standardised methods were used to transcribe and analyse the content of speech and characteristics such as pauses, overlap, stress, intonation, pace and non-verbal communication.^20^ This paper contains simplified transcripts with names and locations changed. Numbers in brackets denote silence in seconds. We coded the following.

- Clinicians’ questions as:
  - (i) open or closed
  - (ii) inviting a yes or no response^20^
- Patients’ responses as:
  - no self-harm response
  - self-harm response
  - ‘non-answer’ that did not provide an answer to the question, e.g. ‘I don't know’^21^
  - resisting the question or an assumption in the question, e.g. ‘I'd like to say no but that's one of those things that – I – I don't think I could guarantee it’.^22,23^
- How the question affected the patient's response. As previous work has shown that questions bias patients’ responses, we used Fisher's exact test to test the hypothesis that no-inviting questions were more likely to elicit ‘no self-harm’ responses.

In the videos, patients did not always show conviction or certainty in responses that claimed ‘no suicidal ideation’ or ‘no self-harm’. Hence, we identified (a) statements indicating lack of knowledge or certainty (e.g. ‘I don't know’), and (b) signs of patient disengagement, which could indicate difficulty or reluctance to disclose more sensitive information. This included flat prosody, quiet or slow speech relative to the patient's typical speech, minimal lexical responses (e.g. ‘No. [2 s]’), averting gaze, flat or unchanging facial expression, and physically turning away from the clinician.^24^ Disengagement was classified as ≥3 markers of disengagement.

### Findings

There were 55 initial questions about current thoughts or feelings of self-harm (*N* = 19), plans to self-harm (*N* = 13), the potential for future self-harm (*N* = 13), predicting risk of future self-harm (*N* = 5) and being okay and/or keeping safe (*N* = 5).

#### Questions were predominantly closed questions to which patients disclosed minimal information

In 84% of cases, clinicians started by asking a closed yes/no question (*N* = 46/55). However, in 16% cases (*N* = 9/55), initial questions were open questions, starting with ‘what/when/how’, inviting the patient to provide a longer response (Table 1).

**Table 1:** Examples of closed and open questions about suicidal ideation/self-harm

Closed question	Open question
Are you feeling like ending your life?	How do you feel about ending your life as we speak now?
Do you still have any thoughts of wanting to end things?	Where are you now with your thoughts of wanting to end your life?
Do you think there's any chance of you doing something else to harm yourself?	What do you think the risks are of something like this happening again?

Closed questions ask patients to choose yes or no, and are designed to constrain responses. Patients typically provided minimal answers followed by silence and did not elaborate on their experiences (*N* = 33/46, 72%). For example:

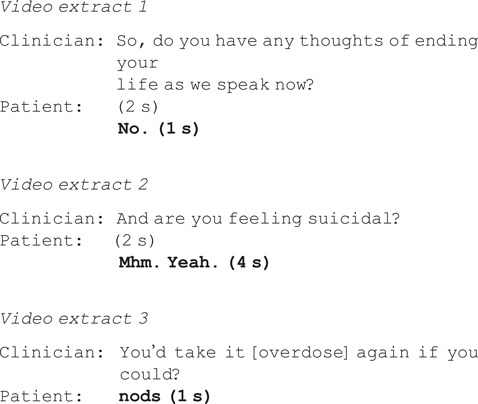


By contrast, open questions elicited longer information-rich responses, with patients frequently describing conflicting or ambiguous thoughts.

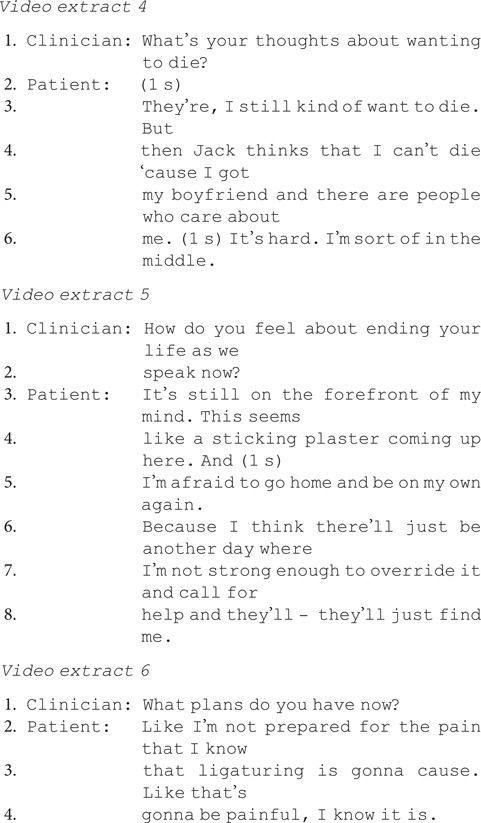


#### Questions were frequently no-inviting questions, which limit patient disclosure

##### No-inviting and yes-inviting questions

As well as inviting minimal responses, closed questions also invite either a yes or a no answer.^12^ Of 46 closed questions, more than half (54%, *N* = 25/46) invited a no-self-harm response, with fewer than half (46%) inviting a yes-self-harm response. No-inviting questions used the negative polarity markers any/ever/at all (Table 2). Negative polarity items are only used in negative statements (e.g. ‘I don't have any plans.’) and not in positive statements (e.g. ‘I have any plans.’).

**Table 2:** No-inviting and yes-inviting questions

**Invites ‘no’ response**	Are you having any thoughts that you'd be better off not here? Do you have any thoughts of harming yourself at the moment? Do you ever kind of plan anything to hurt yourself?
**Invites ‘yes’ response**	So: are you thinking about ending your life at the moment? Are you feeling like ending your life? Are you feeling suicidal?

##### No-inviting questions limit disclosure of self-harm

Patients were significantly less likely to disclose self-harm in response to no-inviting questions, with an 8% disclosure rate (*N* = 2/26) compared with the 65% disclosure rate in response to yes-inviting questions (*N* = 12/20, *P* < 0.0001 Fisher's exact test two-tailed) (Table 3).

**Table 3:** Disclosure rate or ‘no-inviting’ versus ‘yes-inviting’ questions

	Non-answer or no-self-harm response	Yes-self-harm response
**No-inviting question**	92% (*N* = 24/26)	**8% (*N* = 2/26)**
**Yes-inviting question**	35% (*N* = 7/20)	**65% (*N* = 13/20)**

There is further evidence on how patients respond to no-inviting questions demonstrating that such questions are highly problematic for disclosing suicidal ideation and/or self-harm: patients often delayed their response, qualified or downplayed their response, looked away and provided minimal information. These features mark difficulty responding.^25^ For example, in extract 7, there are long delays (lines 3, 5), the patient breaks eye contact (line 3) and eventually confirms (‘I do’) but downplays the extent (‘a few’) and does not describe his ideas or plans in any detail.

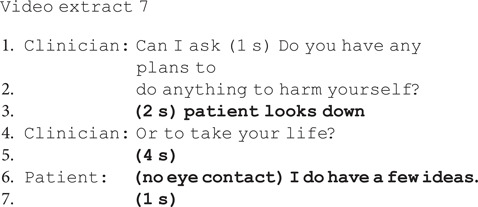


Second, when patients did not disclose suicidal ideation or self-harm, they more frequently showed signs of disengagement and lack of conviction after no-inviting questions. For example, after being asked if she will be okay overnight, the patient in extract 8 confirms but immediately shows signs of disengagement: breaking off eye contact, looking at the floor and nodding silently.^24^ Patients gave a disengaged response 35% of the time (*N* = 7/20) when giving a no-problem answer to a no-inviting question versus 11% of the time (*N* = 1/9) when giving a no-problem answer to a yes-inviting question.

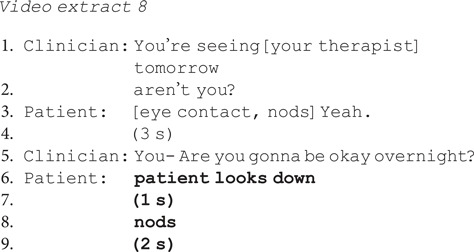


### When asked to predict future thoughts, actions and safety, patients resisted the assumption they could predict the future

Practitioners frequently asked patients about the future: this included their future suicidal thoughts or actions, whether they would repeat self-harm and whether they could keep themselves safe (*N* = 19/55, 35%) (Table 4).

**Table 4:** Questions about the future

**Predict future thoughts/actions**	If you went home now. Would you do anything to harm yourself? Do you think you might have feelings of hurting yourself again?
**Predict risk of (repeat) self-harm**	What do you think are the risks of something like this happening again? Do you think there's any chance of you doing something else to harm yourself?
**Predict future safety**	Does it feel like you'll be able to keep yourself safe? If you were to go home. Do you think that you could keep yourself safe?

Again, there was a bias with 63% (*N* = 12) of these questions inviting the patient to confirm they would not be at risk or could keep themselves safe. Asking for a prediction is different to asking patients whether they currently have plans or thoughts about ending their life (e.g. ‘Do you have any plans to do this again?’).

Patients found these questions particularly difficult to answer, as evidenced by substantial delay and resisting the implication that they could predict future self-harm in the following ways.

- Explicitly claiming lack of knowledge:

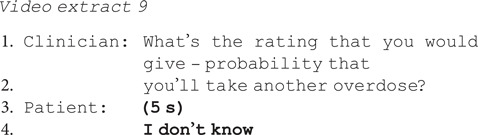
- Hedging their response using ‘I think’, false starts and cut-off words (‘My-’) and downgrading their certainty:

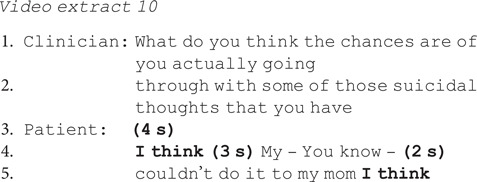

Patients also emphasised the difficulty of assessing the likelihood of future suicidal acts when this was something they did not want, e.g. in extract 11 the clinician asks if the patient thinks he will attempt suicide again.

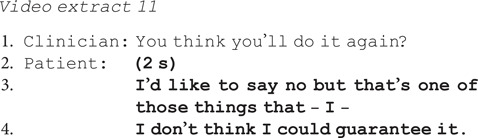


Instead of giving a straightforward yes or no as invited by the question, the patient states ‘I'd like to say no’. He then gives a hedged answer, citing his uncertainty ‘I don't think I could guarantee’ and difficulty predicting whether he will attempt suicide.

By contrast, there are three instances where a clinician asks about the future without asking the patient to predict their future actions. In extract 12, the clinician asks whether the patient is scared she may hurt herself. She immediately confirms, without hesitation, backpedalling or uncertainty, showing no difficulty in making this disclosure.

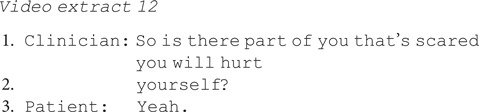


### Half of closed questions had a narrow timeframe or were explicitly tied to possible discharge

Some questions were also temporally constrained with a narrow timeframe (18 of 46 closed questions), as follows:

- nine of 19 questions about *current thoughts/feelings* of self-harm, e.g. ‘So are you thinking about ending your life **at the moment**?’ ‘Do you have any plans of ending your life **once you leave here**?’
- four of 13 questions about *plans to self-harm*, e.g. ‘Have you got any plans **at the moment** to take another overdose or harm yourself?’
- one of 13 questions about the *potential for future self-harm*, e.g. ‘Where are you at **over the next twenty-four hours** about hurting yourself?’
- two of five questions *predicting risk of future self-harm*, e.g. ‘And so do you think there's any chance of you doing something else to harm yourself **today tomorrow?**’
- two of five questions about *being okay*, e.g. ‘Are you gonna be okay **overnight?**’

In addition, towards the end of the assessment, some questions about future self-harm were explicitly tied to possible discharge (6/46 closed questions):

- two of 13 questions about *plans to self-harm*, e.g. ‘**If you were to go home**, to do something that would result in your death? Or harm to yourself?’
- two of 13 questions about *potential for future self-harm*, e.g. ‘On a basic level **if you went home now,** would you do anything to harm yourself?’
- two of five questions about *keeping safe*, e.g. ‘**If you were to go home** do you think that you could keep yourself safe?’

This placement indicates to the patient that their answer is tied to discharge, which is likely to affect how patients respond if they wish to be discharged.

## Discussion

Across the psychosocial assessment, there is a bias towards not uncovering thoughts and plans of self-harm through the cumulative effects of leading questions that invite a no response, their narrow timeframe and tying questions to possible discharge. First, 84% of questions were closed yes/no questions inviting minimal yes/no responses. Second, more than half were leading questions inviting a ‘no’ self-harm answer. When asked no-inviting questions, patients disclosed self-harm in only 8% of cases compared with 65% when asked a yes-inviting question. When answering ‘no’ (e.g. no thoughts or plans of suicide) to no-inviting questions, patients often showed signs of disengagement and lack of conviction (35%). Third, approximately one-third of questions asked patients to predict whether they would harm themselves in the future. Patients showed difficulty responding to these questions, resisting the assumption that they could predict future thoughts or actions, which is underpinned by patients’ criticisms of these questions, including ‘guaranteeing safety’ questions. Finally, half of closed questions had a narrow timeframe (e.g. ‘at the moment, overnight, today, tomorrow, when you leave the emergency department’) or were explicitly tied to possible discharge.

Suicide risk assessment is a multifactorial process, and assessment of self-harm is just one component.^26^ Nonetheless, these findings may help to shed light on the ‘low-risk paradox’, i.e. that patients are usually judged as low or no risk prior to death by suicide^27^ and ‘deny suicidal ideation’ even in the days and weeks before death.^3,4^ Our findings highlight that communication is structured in a way that makes it difficult for patients to disclose self-harm. Clinicians are unaware that a subtle difference in the wording of questions biases the patient's response with the items ‘any, ever, at all’ making questions no-inviting. The narrow timeframes demonstrate that clinicians are focusing on short time periods, typically up to 24 h. At the same time, they are tying patient responses to discharge, i.e. if the patient states they have no immediate thoughts or plans they are safe to discharge. Given that most patients report wanting to leave the emergency department after long waits to be seen in the main emergency department before being referred and then undergoing a psychosocial assessment, there is a risk of patients saying what they need to say so they can leave, i.e. they are not thinking about or planning self-harm. As a whole, these findings shed light on the National Confidential Inquiry findings that of the 17 people who die by suicide each day in the UK, four out of five are judged to be at low or no risk of suicide.

These findings are consistent with studies in out-patient community mental healthcare and general practice, where professionals also mostly asked no-inviting questions.^14,15^ In suicide risk assessment, no-inviting questions are compounded by patient shame, stigma and the consequences of disclosing self-harm (e.g. hospital admission). Concurrent feelings of wanting to live and die and fluctuating feelings about the ability to manage suicidal thoughts can also make these questions difficult to answer. On the other hand, when clinicians asked open questions, patients disclosed more information, particularly around inconsistent or ambiguous feelings of self-harm. Clinician judgement about the likelihood of self-harm could inform how practitioners design their questions. In conversation analysis, this process of tailoring our talk for the person we are speaking to is known as ‘recipient design’. Although this may contribute to how questions are designed, in a previous study we found that practitioners were not more likely to ask a yes-inviting question of patients who self-reported more suicidal thoughts and/or behaviour^14^.

No-inviting questions are not just a feature of suicide risk assessment but a feature of healthcare questioning more widely. Doctors systematically ask questions inviting the ‘best case’ answer (e.g. ‘No pain?’); this is known as the principle of optimisation.^12^ In a randomised controlled trial, doctors who asked ‘Do you have *some* other concerns you would like to discuss?’, inviting a yes, versus ‘Do you have *any* other concerns you would like to discuss?’, inviting a no, were significantly more likely to elicit and reduce unmet concerns when using yes-inviting questions.^13^

Emergency department psychiatric liaison clinicians are faced with increasingly limited options for treatment (e.g. few in-patient beds, long waiting lists for referrals). If a person ends their life, clinicians may be called to a coroner's court if a person deemed at risk does not receive adequate treatment. Anecdotally, clinicians report defensive practice and can experience ‘moral injury’ when they are expected to work in ways that contradict their moral compass.^28^ This includes making recommendations (e.g. discharge) because of limited resources as opposed to their clinical judgement. This can manifest in burnout, clinician turnover, or developing a more detached or even callous approach to care as a defence mechanism against the system's impact on their practice.

Nonetheless, our findings have clear clinical implications. Patients are more likely to disclose self-harm when asked yes inviting (e.g. Are you feeling suicidal?) questions. No inviting questions typically use the words ‘any’, ‘ever’ or ‘at all’ (e.g. ‘any thoughts’, ‘any plans’) or a negative declarative format (e.g. ‘You're not planning on doing it again?’). Open questions e.g. ‘How do you feel about ending your life as we speak now?’, ‘What are your thoughts about wanting to die?’ yield rich responses on the type and strength of thoughts and reasons the patient would not (re)attempt self-harm. Categorising self-harm thoughts and/or plans as either yes or no is problematic, as most people have ambivalent feelings. Guidelines and training should highlight how subtle differences in the wording of questions significantly influence patient disclosure of suicidal ideation and self-harm. For real change, there must also be an understanding of how clinicians protect themselves and cope with the challenges of underresourcing,^28^ along with adequate supervision and training.^29^

Data were collected in one emergency department, which may limit the generalisability of the findings. However, the findings are consistent with communications about self-harm in secondary and primary care. Around three-quarters of clinicians and just under 60% of patients consented: those consenting may not be representative of clinicians and patients. As is typical of many studies, patient participants were mostly White British (*N* = 21/45) and 60% were female. Further research with minority ethnic groups, different socioeconomic groups and other marginalised groups to look at assessments in these groups in more detail would be important to understand whether communication differs and, if so, how it differs. We focused on initial questions as they influence the trajectory of further enquiry. We did not focus on caregiver communication, which was beyond the scope of this analysis. This is the first detailed analysis of video-recorded communication about self-harm in the emergency department. Conversation analysis allowed us to analyse non-verbal communication and to identify how subtle differences in question design affect patient disclosure.

In conclusion, there is a bias towards not uncovering self-harm thoughts and plans in the emergency department. Closed no-inviting questions and asking patients to predict the future are common and deter disclosure. Other problematic aspects are narrow timeframes and tying questions to possible discharge. Open questions and yes-inviting questions facilitate disclosure. Asking patients how they feel about the future rather than asking them to predict whether they will harm themselves also facilitates disclosure.

## Data Availability

Participant data will not be made available owing to ethical restrictions.
